# *CYP3A* and *CYP2B6* Genotype Predicts Glucose Metabolism Disorder among HIV Patients on Long-Term Efavirenz-Based ART: A Case-Control Study

**DOI:** 10.3390/jpm12071087

**Published:** 2022-06-30

**Authors:** Wondmagegn Tamiru Tadesse, Eulambius Mathias Mlugu, Workineh Shibeshi, Wondwossen Amogne Degu, Ephrem Engidawork, Eleni Aklillu

**Affiliations:** 1 Department of Pharmacology and Clinical Pharmacology, School of Pharmacy, College of Health Sciences, Addis Ababa University, Addis Ababa P.O. Box 9086, Ethiopia; wondmagegn.tamiru@aau.edu.et (W.T.T.); workineh.shibeshi@aau.edu.et (W.S.); ephrem.engidawork@aau.edu.et (E.E.); 2 Division of Clinical Pharmacology, Department of Laboratory Medicine, Karolinska Institute, Karolinska University Hospital-Huddinge, 14186 Stockholm, Sweden; mlugusonlove@gmail.com; 3 Department of Pharmaceutics and Pharmacy Practice, School of Pharmacy, Muhimbili University of Health and Allied Sciences, Dar es Salaam P.O. Box 65014, Tanzania; 4 Department of Internal Medicine, School of Medicine, College of Health Sciences, Addis Ababa University, Addis Ababa P.O. Box 9086, Ethiopia; wonamogne@yahoo.com

**Keywords:** antiretroviral therapy, efavirenz, glucose metabolic disorder, HIV, pharmacogenetic variation, *CYP3A5*, *CYP2B6*, genotype, Ethiopia

## Abstract

Long-term antiretroviral treatment (cART) increases the risk of glucose metabolism disorders (GMDs). Genetic variation in drug-metabolizing enzymes and transporters may influence susceptibility to cART-associated GMDs. We conducted a case-control study to investigate the association of pharmacogenetic variations with cART-induced GMDs. A total of 240 HIV patients on long-term efavirenz-based cART (75 GMD cases and 165 controls without GMDs) were genotyped for CYP3A4*1B, CYP3A5 (*3,*6), CYP2B6*6, UGT2B7*2, *ABCB1* (*c.3435C>T*, *c.4036A>G*), and *SLCO1B1* (*1b, *5). GMD cases were defined as the presence of impaired fasting glucose, insulin resistance, or diabetes mellitus (DM). Case-control genotype/haplotype association and logistic regression analysis were performed by adjusting for age, sex, and BMI. The major *CYP3A* haplotype were CYP3A5*3 (53.8%), CYP3A4*1B (17.3%), combinations of CYP3A4*1B, and CYP3A5*6 (10.9%), and *CYP3A* wild type (7%). CYP3A5*6 allele (*p* = 0.005) and CYP3A5*6 genotype (*p* = 0.01) were significantly associated with GMD cases. Multivariate analysis indicated *CYP3A* haplotype as a significant predictor of GMD (*p* = 0.02) and IFG (*p* = 0.004). CYP2B6*6 significantly predicted DM (*p* = 0.03). *CYP3A* haplotype and CYP2B6*6 genotype are independent significant predictors of GMD and DM, respectively, among HIV patients on long-term EFV-based cART.

## 1. Introduction

The introduction of combination antiretroviral therapy (cART) significantly improved survival and health-related quality of life among people living with HIV (PLWH) [1,2]. Efavirenz (EFV)-based cART is among the first-line antiretroviral regimen to treat HIV recommended by the World Health Organization [1]. Before its takeover by dolutegravir, EFV served as the backbone of the preferred first-line antiretroviral regimen due to its clinical, operational, and programmatic benefits.

Various observational and clinical trial studies link EFV use with a range of treatment-associated adverse events manifested depending on the duration of therapy. While liver enzyme abnormalities [3,4,5,6] and neuropsychological manifestations [7,8] can occur during early treatment, HIV patients on long-term antiretroviral therapy are at an increased risk of developing metabolic derangements, including lipid dystrophy and glucose metabolism disorders (GMDs) [9,10,11]. Indeed, a higher risk of GMDs among HIV patients on EFV-based cART than on atazanavir/ritonavir-based cART has been reported recently [11].

Several factors determine individuals’ susceptibility to cART-associated adverse events [12,13], including comorbidities and concomitant medications, host-genetic factors, and population variation [14,15,16]. Genetic variations in genes coding for drug-metabolizing enzymes and transporter proteins can alter plasma concentration and drug exposure, ultimately affecting treatment outcomes. Efavirenz is metabolized by genetically polymorphic CYP enzymes expressed in the liver. It is primarily metabolized to hydroxylated metabolites by CYP2B6, though other CYPs, including CYP3A4/5, CYP1A2, and CYP2A6, also play a minor role [17]. Genetic variations in drug-metabolizing enzymes affect EFV plasma exposure [15,18,19], thereby altering treatment outcomes [4,8].

Pharmacogenetic variation in relevant EFV metabolizing enzymes and transporter proteins may be linked with metabolic derangements and result in clinical conditions, including treatment-associated adverse events. Indeed, the association of *CYP2B6* defective variant allele and higher plasma efavirenz exposure with liver enzyme abnormalities [3,4,5,20] and neuropsychological manifestations is reported [7,8,20].

Accumulating evidence in the literature indicates that long-term efavirenz-based cARTs are associated with GMDs in PLWH [11,21,22]. The importance of pharmacogenetic variations for cART-induced liver and CNS toxicities is well explored. Inter-individual variations in drug metabolic capacity due to genetic variations of drug-metabolizing enzyme and transporter genes may play a role in the predisposition to GMDs (insulin resistance (IR) or impaired fasting glycemia (IFG)) in antiretroviral therapy (ART).

No studies have been conducted to assess the association of GMDs with genetic variations in enzymes and transporter proteins involved in the disposition of EFV. Pharmacogenetic association studies are important to identify genetic biomarkers that predict specific ADRs associated with ARVs. Identifying risk factors of EFV-based cART-associated GMD is imperative in Africa, where HIV remains a significant public health problem. Here, we assessed the association of common functional variant alleles in relevant drug-metabolizing enzymes (*CYP3A4*, *CYP3A5*, *CYP2B6*, and *UGT2B7*) and transporters (*ABCB1* and *SLCO1B1*) with cART-associated GMDs.

## 2. Materials and Methods

### 2.1. Study Setting Design and Participants

The study setting was the HIV clinic of Tikur Anbessa Specialized Hospital (TASH), College of Health Sciences, Addis Ababa University, Addis Ababa, Ethiopia. TASH, being the largest tertiary level teaching and referral hospital in Ethiopia, serves about 500,000 patients each year through its various specialized clinical services. The hospital receives referred patients from different corners of the country and the city, providing an opportunity to recruit study participants representing a heterogeneous mix of different ethnic populations and socioeconomic statuses of the country. The HIV clinic at TASH coordinates various activities encompassing HIV/AIDS prevention, patient care, and ART services for patients through scheduled follow-ups.

The study design was a case-control genotype association study, and the study population is described in our previous cross-sectional study [11]. In brief, a total of 240 participants on long-term EFV-based ART regimens with complete genotyping and GMD data participated in this study. The inclusion and exclusion criteria were stated in detail in our previous publication [11]. In brief, PLWH on EFV-based cART at least for one year with age ≥ 18 years were included, while PLWH diagnosed with DM, pregnancy, cancer, renal disease, liver disease, uncontrolled hypertension, or heart failure were excluded from this study. Additionally, patients on concomitant treatment with antipsychotics, anti-cancer agents, anti-TB, corticosteroids, hormonal agents, and antidiabetic agents were excluded.

Whole blood was collected after overnight fasting (8 to 12 h). Serum fasting blood glucose (FBG), insulin, and lipid profiles were determined. Homeostatic Model Assessment for Insulin Resistance (HOMA-IR) was calculated by using the following equation [23].

$$\mathrm{HOMA}-\mathrm{IR}=\frac{\left[\mathrm{Fasting}\;\mathrm{insulin}\;\left(\frac{\mu U}{\mathrm{mL}}\right)\times \mathrm{Fasting}\;\mathrm{glucose}\left(\frac{\mathrm{mmol}}{L}\right)\right]}{22.5}$$

### 2.2. GMD Case Definitions

The following operational definitions were used [11].

1. DM was defined as a fasting glucose level of 126 mg/dL or higher.
2. IFG was defined as a fasting glucose level between 110 and 125 mg/dL.
3. IR was diagnosed by either a homeostasis model assessment insulin resistance (HOMA-IR) value of ≥3.8, Fasting plasma insulin of ≥20 μU/mL, or fasting glucose/insulin ratio of ≥4.5′.
4. GMDs were defined as the presence of IFG, IR, or DM.

Controls were those individuals with normal fasting serum glucose, HOMA-IR, or serum insulin level based on the above-stated cut-off values as stated by Tadesse et al. [11]. The cases were those individuals with at least one diagnosis with insulin resistance, impaired fasting glycemia, or diabetes mellitus. The case and control groups were matched to each other in terms of age, duration since cART start, duration on current cART, weight, waist circumference, and BMI. Out of 240 participants, 75 cases and 165 controls were recruited in this study.

### 2.3. Genotyping

Whole blood samples were collected for genotype analysis in EDTA coated vacutainer tube and inverted 8–10 times for thorough mixing of EDTA. The samples were then transferred to cryotubes and stored at −80 °C until genotyping analysis. Genomic DNA was isolated from whole-blood samples using QIAamp DNA MidiKit (QIAGEN GmbH, Hilden, Germany) according to the manufacturer’s instructions. Purity and quantity of DNA were assessed using NanoDrop 2000 (Thermo Scientific, Saveen Warner, Sweden). Allelic discrimination assay was performed using TaqMan drug metabolism genotyping assay (Applied Biosystems, Foster City, CA, USA).

Functional variant alleles in enzymes and transport proteins relevant for disposition of EFV based cART were selected for genotyping [15,17]. The variant alleles were CYP3A4*1B, CYP3A5*3, CYP3A5*6, CYP2B6*6, UGT2B7*2, *ABCB1* (*c.3435C>T* and *c.4036A>G*), and *SLCO1B1* (*1B, *5). Genotyping was performed using TaqMan^®^ allele-specific PCR (Applied Biosystems Genotyping Assays) with the following ID numbers for respective SNPs: C__7586657_20 for *ABCB1c.3435C>T*, C_11711730_20 for *ABCB1c.4036A>G*, C__7817765_60 for *CYP2B6 c.516G>T* (CYP2B6*6), C__30720663_20 for *UGT2B7 −327G>A* (UGT2B7*2b, *2c, *2d, *2f), C_9440184_20 for UGT2B15*4, C__26201809_30 for CYP3A5*3 (*6986A>G*), C__30203950_10 for CYP3A5*6 (*14690G>A*), C_1837671_50 for CYP3A4*1B, C_1901697_20 for SLCO1B1*1B, C_30633906_10 for SLCO1B1*5. The 7500 Real-Time PCR system (Applied Biosystems) was used for genotyping. The final volume was 10 μL for each reaction, consisting of 9 µL of TaqMan Universal PCR Master Mix^®^ (Applied Biosystems, Waltham, MA, USA), DNA/RNA free water, TaqMan 20× drug-metabolism genotyping assay mix (Applied Biosystems), and 1 µL of genomic DNA. The PCR conditions were as follows: an initial step at 60 °C for 30 s, hold-stage at 95 °C for 10 min and PCR stage for 40 cycles step 1 with 95 °C for 15 and step 2 with 60 °C for 1 min and after read-stage with 60 °C for 30 s.

### 2.4. Statistical Analysis

Hardy–Weinberg equilibrium was assessed by chi-square test for each SNP to determine any differences between observed and expected genotype frequencies. Statistical analyses were performed using Statistical Package for Social Sciences (SPSS) version 26 (IBM^®^ SPSS^®^ Statistics, Chicago, IL, USA) and Haploview version 4.2 (Broad Institute, Cambridge, MA, USA). Baseline sociodemographic and laboratory parameters were described as means and standard deviation (SD) or medians, and interquartile range (IQR) for continuous variables and proportions for categorical variables.

Haploview version 4.2 was employed to determine the association and case-control analysis of genotypes and haplotypes. The association of GMDs with genetic variants was determined through logistic regression analysis. Univariate followed by a multivariate logistic regression model was used to determine the association of GMD with genotype variants after adjusting for factors such as age, sex, and BMI among the groups. Genotypes with a univariate analysis of *p* < 0.2 were entered in the multivariate analysis. A stepwise conditional-backward analysis was employed when three or more genotypes meet the stated entry criteria for multivariate analysis. When only one or two genotypes meet the entry criteria, a direct enter method (without a stepwise) was used in the multivariate analysis. *p*-values of <0.05 were considered statistically significant. The regression coefficient (β), crude odds ratio (COR), and adjusted odds ratio (AOR) were recorded from univariate and multivariate logistic regression.

### 2.5. Ethical Consideration

Ethical clearance was obtained from the Institutional Review Board of College of Health Sciences, Addis Ababa University (Protocol No. 019/19/SoP) and National Ethical Review Committee, Ministry of Science and Higher Education of Ethiopia (Ref. No. MoSHE/RD14.1/9324/20). Written informed consent was obtained from each study participant after fully explaining the purpose and nature of all procedures used.

## 3. Results

### 3.1. Characteristics of Study Participants

A comparison of sociodemographic, clinical, and biochemical parameters between HIV patients who developed GMD (cases) versus those who did not develop GMD (controls) is presented in Table 1. The highest mean age (46.6 ± 1.3 years) was recorded in the case group. Unlike the case group, the distribution of males (21.7%) and females (78.3%) was disproportional among the control group. The other characteristics, such as weight, BMI, and duration on cART, did not show a significant difference between the case and the control groups (Table 1). Comparative clinical laboratory data for lipid measurements, fasting serum glucose, fasting serum insulin, and HOMA-IR values are shown in Table 1. A significant mean difference (*p* < 0.001) was observed in the biochemical markers of GMD between the control and the case groups (Table 1).

### 3.2. Genotype, Allele, and Haplotype Frequencies

Comparison of CYP3A4*1B, CYP3A5*3, CYP3A5*6, CYP2B6*6, UGT2B7*2, *ABCB1 c.3435C>T*, *ABCB1c.4036A>G*, SLCO1B1*1B, and SLCO1B1*5 genotype and allele frequencies between control and case groups are depicted in Table 2. All genotype frequencies were in accordance with Hardy–Weinberg equilibrium (HWE) (*p* > 0.05).

**Figure 1:**
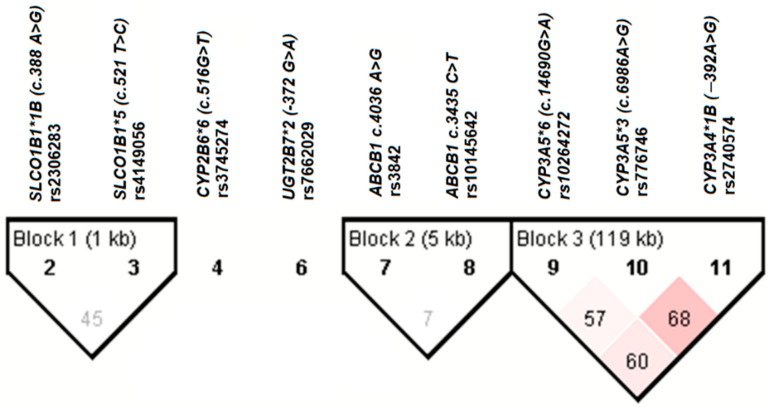
Linkage disequilibrium (LD) plot of the studied genotype variants among Ethiopian PLWH on EFV-based cART. The values in the diagonal square boxes represent the respective observed D′ values of the pair-wise LD relationship. The red color gradient indicates higher LD values while the white represents lower LD values (D′ 1–0).

The variant allele frequency CYP3A5*6 was significantly higher among GMD cases than in the control groups (0.41 versus 0.24) (*p* = 0.005). The linkage disequilibrium (LD) analysis demonstrated a linkage among the three *CYP3A* valiant alleles, and the relatively stronger linkage was between CYP3A4*1B and CYP3A5*3 (Figure 1). The major *CYP3A* haplotype was CYP3A5*3 alone (53.8%), followed by CYP3A4*1B alone (17.3%) and then by CYP3A5*1B linkage with CYP3A5*6 (10.9%) (Table 3). Based on Haploview case-control analysis, the CYP3A5*6 allele was significantly associated with IFG, IR, and overall GMD, while the CYP2B6*6 allele was significantly associated with DM (not shown in tables).

### 3.3. Genotype/Haplotype Association with GMD

Based on Haploview case-control analysis, 3A4*1B + 3A5*6 haplotype combinations of *CYP3A* showed a significant association with GMD (*p* = 0.04) (Table 3). The multivariate analysis revealed that the 3A4*1B + 3A5*3 + 3A5*6 (*1B/*3/*6) haplotype combination of CYP3A was found to be an independent predictor of GMD when the wildtype and the *1B haplotype carriers used as a reference group (overall *p* = 0.02) (Table 4). Among carriers of CYP3A4*1B + 3A5*3 + 3A5*6 haplotype, the odds of experiencing GMD were about 2.2-fold higher (AOR = 2.2; 95% CI 1.0–4.8), *p* = 0.04) than carriers of the wildtype (with two functional alleles) or CYP3A5*3 alone haplotype combinations.

### 3.4. Genotype/Haplotype Association with IFG

As depicted in Table 3, the Haploview case-control analysis revealed that 3A4*1B + 3A5*6 haplotype combinations of *CYP3A* demonstrated a significant association with IFG (*p* < 0.01), whereas the multivariate analysis (Table 4) showed that the overall *CYP3A* haplotypes were significantly associated with IFG (*p* = 0.004). In alternative multivariate analysis, the odds of developing IFG was 2.8-times higher among 3A4*1B + 3A5*3 + 3A5*6 haplotype carriers compared to the wildtype or CYP3A5*3-alone haplotypes carriers (AOR = 2.8, 95% CI 1.1–7.1, *p* = 0.04).

### 3.5. Association with Insulin Resistance

Based on Haploview case-control analysis, carriers of CYP3A4*1B + 3A5*6 haplotype combination of *CYP3A* locus showed a significant association with the incidence of IR (*p* = 0.04) (Table 3). *CYP3A* haplotypes showed marginal association compared to the wildtype and the *1B haplotype carriers (*p* = 0.06) (Table 5). In alternative multivariate analysis (not shown in tables), 3A4*1B + 3A5*3 + 3A5*6 haplotype combination predicted the incidence of IR. The odds of IR risk were 2.6 for 3A4*1B + 3A5*3 + 3A5*6 haplotype carriers (AOR = 2.6; 95% CI 1.0–6.8, *p* = 0.05) compared to the wildtype or CYP3A5*3-alone haplotype combinations. In addition, the T allele of *ABCB1 c.3435T* showed a significant 60% protection of IR (AOR = 0.4, 95% CI 0.2–0.9, *p* = 0.04) relative to the wildtype *ABCB1 c.3435* C allele (Table 5).

### 3.6. Association with DM

As depicted in Table 5, CYP2B6*6 c.516G>T genotype significantly predicted the incidence of DM among study participants on EFV-based cART. Based on multivariate regression analysis, carriers of CYP2B6*6 alleles were at a significantly higher risk of DM by 4-fold than the wildtype carriers (AOR = 4.0; CI 95% 1.1–14.5; *p* = 0.03). No significant association of DM with the other genotypes was observed.

## 4. Discussion

Long-term ART is associated with several metabolic adverse effects, including GMDs (i.e., IR, IFG, or Type 2 diabetes). Long-term efavirenz-based cART-associated metabolic derangements, including GMD, are likely due to mitochondrial toxicity [9]. Growing evidence implicates the role of efavirenz in energy metabolism, mitochondrial function, and other cellular processes involved in oxidative stress [24,25]. Efavirenz exposure lowers glucose uptake and alters bioenergetic cell profiles in human cell lines through inhibition of SLC2A1, which mediates cellular glucose uptake [26]. This result in reduced glucose metabolism in immune cells, suppressing immune cell activation. The primary metabolite of efavirenz, 8-Hydroxy-efavirenz, stimulates the glycolytic flux in cultured rat astrocytes [27]. Association of high plasma efavirenz concentrations with high plasma fasting lipid concentrations and glucose concentrations in South African HIV patients treated with efavirenz-based ART is reported [28]. In our previous study, a higher prevalence of GMDs (27.6%) was reported among HIV patients on a long-term EFV-based regimen [11].

Altered efavirenz metabolism and disposition due to pharmacogenetic variation may influence susceptibility to treatment-induced glucose metabolic disorder. So far, no literature investigated the association of GMDs with functional variant alleles in genes relevant for EFV disposition among PLWH on long-term treatment. Therefore, in this case-control study, we investigated the association of genotype/haplotype variants with the incidence of GMDs among Ethiopian PLWH on EFV-based cARTs. The main finding of this study includes (1) *CYP3A* haplotype, particularly CYP3A4*1B + 3A5*3 + 3A5*6 allele combinations, which were a significant predictor of IR, IFG, and overall GMD, and (2) CYP2B6*6 is significantly associated with the risk of developing DM in EFV-based cART. To our knowledge, this is the first study to explore the importance of pharmacogenetic variation for susceptibility to antiretroviral treatment-induced GMD.

There was no statistically different mean in the age, duration of time since cART start, period on the current cART type, weight, waist circumference, and BMI between the case and the control groups, implying a matching between the case and the control groups in the study. We also showed that cases had significantly elevated HOMA-IR, FSG, FSI, and TG levels than controls, indicating the presence of GMDs. The overall observed genotype and allele frequency proportions were similar to the findings of previous studies from Ethiopia [29,30].

Interestingly, the distribution of CYP3A5*6 allele and genotype frequencies between cases and controls were significantly different, being higher in cases than in controls (Table 2). *CYP3A* haplotype combination containing 3A5*6 significantly predicted the incidence of IR, IFG, and overall GMD than the wildtype or CYP3A5*3 alone carriers (Table 3). case-control analysis showed the association of CYP3A4*1B and *CYP3A5* variants with the incidence of GMD, particularly when 3A4*1B and 3A5*6 variants co-occur. Though patients on antiretroviral therapy were not stated to be included, a Japanese case-control study indicated that *CYP3A4* (*13989A>G*) polymorphism is associated with the prevalence of type 2 diabetes mellitus [31]. Additionally, another study reported the association of CYP3A4*18B with the incidence of tacrolimus-induced new-onset diabetes while reporting no association of *CYP3A5* variants with new-onset type 2 diabetes in renal transplant recipients [32], but these studies were on study participants with different disease conditions and types of treatment than our study participants that limited direct comparison.

The effect of the CYP3A4*1B genotype on enzyme activity is not fully understood, and data are inconclusive. As a proximal promoter variant, however, the CYP3A4*1B polymorphisms may influence protein expression more than the catalytic activity. While studies indicated reduced enzyme activity [33], there are also reports of higher expression and enzyme activities among carriers of *1B [34]. Nevertheless, CYP3A4*1B appears to have no or very limited functional impact on CYP3A4 enzyme activity, and variation in the overall CYP3A enzyme activity is mainly influenced by its linkage disequilibrium with *CYP3A5* variant alleles common in the black African population [35,36,37]. CYP3A enzyme activity and genetic variation display wide between-population variation even within black Africans [35]. The major *CYP3A* haplotype in Tanzanians is CYP3A4*1B alone (34.2%), followed by its linkage with CYP3A5*6 (17.6%) and CYP3A5*3 (13.3%) [36]. CYP3A5*7 is absent in Ethiopians. In our study, we found the major *CYP3A* haplotype among Ethiopians being CYP3A4*3 alone (53.8%) and CYP3A4*1B alone (17.3%), followed by CYP3A4*1B linkage with CYP3A5*6 (10.9%) or CYP3A5*3 (6.3%). In the case of CYP3A5*3, as it has reduced activity, it may function better, unlike the null-functioning CYP3A5*6 variant in metabolizing activity. Yet, the CYP3A4*1B + CYP3A5*6 haplotype carriers have reduced active enzymes than carriers of CYP3A5*3 allele or wildtype haplotype.

The higher association of GMD with the 3A4*1B + 3A5*6 haplotype in our study may arise due to the impact of CYP3A5*6 null-functional status. Supporting this assertion, the allele-based case-control analysis (Haploview) consistently showed the association of CYP3A5*6 with GMDs. In general, the implication of the association of GMD with these variants could be due to a defective enzyme function which might result in higher plasma drug exposure linked with the risk of GMDs. Yet, the exact molecular mechanism that links *CYP3A* variants to GMD remains to be explored.

Notably, the CYP2B6*6 genotype significantly predicted the incidence of DM. Similarly, the case-control analysis showed that the CYP2B6*6 allele frequency was significantly higher in the GMD case group than in the control group implying the link between a defective enzyme function with the risk of DM in long-term EFV-based cART. To our knowledge, this is the first finding that showed a statistically significant association of the *CYP2B6* variant allele with the incidence of DM among long-term EFV-based regimens. The CYP2B6*6 genotype determines interindividual variability of plasma efavirenz exposure. In our study, CYP2B6*6 (*c.516G>T*) carriers had a four-fold higher risk of DM as compared to the wildtype carriers. Different from our findings, a previous study did not observe a significant association of CYP2B6*6 with fasting glucose and 2-hour glucose concentration among patients on EFV-based regimens. The authors explained that the small sample size with the genotype data might have limited their study to detect statistically significant genetic associations [28].

In addition, *ABCB1 c.3435C>T* was associated with IR, where the T allele showed a more protective effect than the wildtype. A Turkish case-control study, despite methodological differences from our study, reported that the homozygous mutant of *ABCB1 c.3435C>T* is associated with higher BMI among the control group than in Type 2 DM cases [38], implying a protective role among the case group. Similarly, our study showed a trend that the T allele frequency is slightly higher among control groups than in the cases, although not statistically significant. However, the multivariate analysis revealed that the mutant variants protectively predicted IR. The explanation for this might be that the *ABCB1,* which encodes the efflux transporter P-glycoprotein is involved in the transport of substrates such as sugars, lipids, amino acids, and steroids, apart from their pharmacokinetic role. Functionally, studies suggest that variants with the T allele and the homozygous *ABCB1 c.3435C>T* mutant are linked with reduced expression and functioning of efflux transporters [39]. Thus, SNPs in these genes might alter substrate-related risk factors preventing the occurrence of metabolic conditions such as obesity, one of the predominant risk factors of IR and GMDs, resulting from altered hormonal or physiological biomolecules [40].

In our study, we employed a candidate gene association approach considering variant alleles that may affect the disposition of an EFV-based ART regimen. To our knowledge, for the first time, our study generated evidence of the association between pharmacogenetic variations in drug-metabolizing enzymes and drug transporters relevant for the disposition of antiretrovirals with metabolic disorders such as IR, IFG, and DM among PLWH. The inclusion of insulin and IR parameters as GMD would provide a cumulative prediction of such incidences among PLWH on long-term cART. One of the limitations of our study is that, although in at least 1–4 cases, the control ratio was maintained in most gene variants, the number of participants in the case group was relatively smaller than in the control groups. Our study also focused only on related pharmacokinetic genes and did not consider other SNPs of genes directly involved in carbohydrate metabolism and insulin release. Therefore, we recommend confirmatory studies on a larger sample size with more extended and repeated follow-ups for future studies.

## 5. Conclusions

Our findings suggested that *CYP3A*, *CYP2B6*, and *ABCB1* genotypes predicted treatment-associated GMDs to a varying degree among HIV patients on long-term EFV-based combination antiretroviral therapies. Mainly, the defective functional CYP3A5*6 allele and its haplotype combination (3A4*1B/3A5*3/*3A5*6) were independent predictors of the overall GMDs, IR, and IFG. Intriguingly, the CYP2B6*6 genotype significantly predicted the occurrence of DM (increased risk) on long-term EFV-based cART. Further studies with a large sample size are needed to confirm our findings among PLWH on cART.

## Figures and Tables

**Table 1:** Sociodemographic and biochemical parameters among HIV patients with glucose metabolic disorders (cases) versus without (controls).

Variables		GMD Cases	Control	*p*
Sample (*n*) (case–control ratio)		75	165	(1:2.2)
Gender *n* (%)	Male	40 (52.7)	35 (21.7)	<0.001
	Female	35 (47.3)	130 (78.3)	
Age (years)		46.6 ± 1.3	43.8 ± 0.8	0.06
Duration since HIV confirmed date (month)		141.6 ± 5.9	130.5 ± 3.7	0.10
Cumulative time on cART (month)		129.2 ± 5.3	120.9 ± 3.4	0.19
Time on current cART regimen type (month)		95.3 ± 5.2	92.1 ± 3.1	0.59
Cumulative time on EFV-based 1st-line (month)		104.3 ± 5.0	100.2 ± 3.3	0.49
Time on prior cART regimen types (month)		32.2 ± 4.4	36.2 ± 8.0	0.74
Time on NVP-based 1st-line (Prior to EFV) (month)		24.8 ± 4.0	20.8 ± 3.0	0.43
Weight (Kg)		64.5 ± 1.4	62.7 ± 1.1	0.34
Waist circumference (cm)		34.8 ± 0.6	34.2 ± 0.4	0.37
BMI (Kg/m^2^)		24.1 ± 0.5	24.2 ± 0.4	0.86
Total cholesterol (mg/dL)		205.4 ± 5.1	209.5 ± 14.2	0.85
Triglyceride (mg/dL)		185.0 ± 11.8	136.8 ± 4.3	<0.001
HDL-C (mg/dL)		44.9 ± 2.3	46.8 ± 2.4	0.63
LDL-C (mg/dL)		139.7 ± 18.5	133.4 ± 10.0	0.75
Biochemical markers of GMD	Fasting serum glucose (mg/dL)	117.7 ± 4.9	94.0 ± 0.6	<0.001
	Fasting serum insulin (μLU/mL)	16.9 ± 2.3	7.1 ± 0.3	<0.001
	HOMA-IR	5.3 ± 1.0	1.7 ± 0.1	<0.001

**Table 2:** Genotype and variant allele frequency distribution and association with glucose metabolism disorders (GMD) among PLWH (*N* = 351).

Genotypes	Variants	Control, *n* (%)	Case, *n* (%)	*p*
CYP 3A4*1B (*−392A>G*)	*1/*1	70 (42.4)	28 (37.3)	0.48
	*1/*1B	79 (47.9)	36 (48.0)	
	*1B/*1B	16 (9.7)	11 (14.7)	
CYP3A5*3 c.6986A>G	*1/*1	16 (9.7)	10 (13.3)	0.63
	*1/*3	82 (49.7)	38 (50.7)	
	*3/*3	67 (40.6)	27 (36.0)	
CYP3A5*6 c.14690G>A	*1/*1	126 (76.4)	44 (58.7)	0.01
	*1/*6	36 (21.8)	29 (38.7)	
	*6/*6	3 (1.8)	2 (2.7)	
CYP2B6*6 c.516G>T	*1/*1	81 (49.1)	30 (40.0)	0.42
	*1/*6	70 (42.4)	38 (50.7)	
	*6/*6	14 (8.5)	7 (9.3)	
UGT2B7*2 (*−372G>A*)	*GG*	39 (23.6)	17 (22.7)	0.70
	*GA*	86 (52.1)	36 (48.0)	
	*AA*	40 (24.2)	22 (29.3)	
*ABCB1 c.3435C>T*	*CC*	93 (56.4)	45 (60.0)	0.65
	*CT*	62 (37.6)	24 (32.0)	
	*TT*	10 (6.1)	6 (8.0)	
*ABCB1 c.4036A>G*	*AA*	107 (64.8)	53 (70.7)	0.32
	*AG*	55 (33.3)	19 (25.3)	
	*GG*	3 (1.8)	3 (4.0)	
SLCO1B1*1B c.388A>G	*AA*	22 (13.3)	8 (10.7)	0.84
	*AG*	91 (55.2)	43 (57.3)	
	*GG*	52 (31.5)	24 (32.0)	
SLCO1B1*5 c.521T>C	*TT*	108 (65.5)	49 (65.3)	0.71
	*TC*	49 (29.7)	24 (32.0)	
	*CC*	8 (4.8)	2 (2.7)	
Minor Variant Allele		Allele Frequency		*p*
CYP3A4*1B (*−392A>G*)	*1B	0.58	0.37	0.46
CYP3A5*3 (*c.6986A>G*)	*3	0.9	0.87	0.4
CYP3A5*6 (*c.14690G>A*)	*6	0.24	0.41	0.005
CYP2B6*6 (*c.516G>T*)	*6	0.51	0.6	0.19
UGT2B7*2 (*-372G>A*)	*A*	0.76	0.77	0.87
*ABCB1 c.3435C>T*	*T*	0.44	0.4	0.6
*ABCB1 c.4036A>G*	*G*	0.35	0.29	0.38
SLCO1B1*1B (*c.388A>G*)	*G*	0.87	0.89	0.56
SLCO1B1*5 (*c.521T>C*)	*C*	0.35	0.35	0.99

**Table 3:** GMD case-control analysis of *CYP3A* haplotype using Haploview among HIV patients on long-term EFV-based cART.

*CYP3A* Haplotypes	*CYP3A* Allele Combinations			F (%)	*p*-Values			
	CYP3A4*1B −392A>G	CYP3A5*3 c.6986A>G	CYP3A5*6 c.14690G>A		GMD	IFG	IR	DM
AGG	wt	*3	wt	53.8	0.40	0.20	0.41	0.56
GAG	*1B	wt	wt	17.3	0.90	0.81	0.89	0.27
GAA	*1B	wt	*6	10.9	0.04	0.008	0.04	0.35
GGG	*1B	*3	wt	6.3	0.45	0.17	0.57	0.35
AAG	wt	wt	wt	7.0	0.13	0.99	0.15	0.49
AGA	wt	*3	*6	3.4	0.34	0.32	0.60	0.99

F (%) represent haplotype frequency distribution; level of significance (*p*-values) are based on Haploview case-control analysis for *CYP3A* haplotypes; Wt—wildtype; GMD—glucose metabolism disorder; IFG—impaired fasting glycemia; IR—insulin resistance; DM—diabetes mellitus.

**Table 4:** GMD regression analysis of drug-metabolizing and transporter genotypes with glucose metabolism disorders (GMD) and impaired fasting glycemia (IFG) among HIV patients on long-term EFV-based cART (*n* = 240).

Genotype		GMD among EFV-Based cART						IFG among EFV-Based cART					
		β	COR (95% CI)	*p*	β	AOR (95% CI)	*p*	β	COR (95% CI)	*p*	β	AOR (95% CI)	*p*
*CYP3A* haplotypes (3A4*1B/3A5*3/3A5*6)	*1/*1/*1 or *1B/*1/*1		1	0.02		1	0.02		1	0.004		1	0.004
	*1/*3/*1 or *1B/*3/*1	−0.4	0.7 (0.2, 2.4)	0.56	−0.4	0.6 (0.2, 2.3)	0.5	−0.6	0.6 (0.1, 2.8)	0.47	−0.6	0.6 (0.1, 2.8)	0.47
	*1B/*1/*6 or *1/*3/*6 (any 3A5*6)	0.5	1.6 (0.4, 5.7)	0.49	0.4	1.5 (0.4, 5.6)	0.52	0.7	1.9 (0.4, 9.7)	0.42	0.7	1.9 (0.4, 9.7)	0.42
CYP2B6*6 (*c.516G>T*)	*1/*1		1						1				
	*1/*6 or *6/*6	0.4	1.4 (0.8, 2.5)	0.19	0.4	1.5 (0.9, 2.7)	0.13	0.01	1.0 (0.5, 2.0)	0.97			
UGT2B7*2b (*−327G>A*)	GG		1						1				
	GA or AA	0.1	1.1 (0.6, 2.0)	0.87				0.3	1.4 (0.6, 3.3)	0.49			
*ABCB1 c.3435C>T*	CC		1						1				
	CT or TT	−0.2	0.9 (0.5, 1.5)	0.6				0.2	1.2 (0.6, 2.4)	0.65			
*ABCB1 c.4036A>G*	AA		1						1				
	AG or GG	−0.3	0.8 (0.4, 1.4)	0.38				−0.05	1.0 (0.5, 2.2)	0.9			
SLCO1B1*1B (*c.388A>G*)	AA		1						1				
	AG or GG	0.3	1.3 (0.5, 3.0)	0.56				−0.1	0.9 (0.3, 2.5)	0.84			
SLCO1B1*5 (*c.521T>C*)	TT		1						1				
	TC or CC	0.01	1.0 (0.6, 1.8)	0.99				−0.1	0.9 (0.4, 1.9)	0.77			

COR—crude odds ratios; AOR—adjusted odds ratio.

**Table 5:** Logistic regression analysis of drug-metabolizing and transporter genotypes with insulin resistance (IR) and diabetes mellitus (DM) among HIV patients on long-term EFV-based cART (*n* = 240).

Genotype		IR among EFV-Based cART						DM among EFV-Based cART					
		β	COR (95% CI)	*p*	β	AOR (95% CI)	*p*	β	COR (95% CI)	*p*	β	AOR (95% CI)	*p*
*CYP3A* haplotypes (3A4*1B/3A5*3/3A5*6)	*1/*1/*1 or *1B/*1/*1		1	0.06		1	0.06		1	0.68			
	*1/*3/*1 or *1B/*3/*1	−0.5	0.6 (0.1, 3.1)	0.58	−0.7	0.5 (0.1, 2.6)	0.40	−0.1	0.9 (0.1, 7.5)	0.92			
	*1B/*1/*6 or *1/*3/*6 (any *6)	0.4	1.5 (0.3, 7.8)	0.60	0.2	1.2 (0.2, 6.5)	0.82	−0.7	0.5 (0.05, 5.3)	0.57			
CYP2B6*6 (*c.516G>T*)	*1/*1	0.1	1						1				
	*1/*6 or *6/*6	−0.1	1.0 (0.5, 1.9)	0.9				1.4	4.0 (1.1, 14.5)	0.03	1.4	4.0 (1.1, 14.5)	0.03
UGT2B7*2b (*−327G>A*)	GG		1						1				
	GA or AA	−0.3	0.8 (0.3, 1.7)	0.5				−0.1	0.9 (0.3, 2.9)	0.87			
*ABCB1 c.3435C>T*	CC		1						1				
	CT or TT	−0.6	0.5 (0.3, 1.2)	0.12	−0.8	0.5 (0.2, 0.98)	0.05	−0.2	0.8 (0.3, 2.3)	0.68			
*ABCB1 c.4036A>G*	AA		1						1				
	AG or GG	−0.5	0.6 (0.3, 1.4)	0.25				0.5	1.6 (0.6, 4.5)	0.36			
SLCO1B1*1B (*c.388A>G*)	AA		1						1				
	AG or GG	1.8	5.8 (0.8, 44.0)	0.09	1.9	6.3 (0.8, 49.1)	0.08	−0.5	0.6 (0.2, 2.2)	0.44			
SLCO1B1*5 (*c.521T>C*)	TT		1						1				
	TC or CC	0.2	1.2 (0.6, 2.6)	0.56				0.1	1.1 (0.4, 3.3)	0.8			

COR—crude odds ratios; AOR—adjusted odds ratio.

## Data Availability

All data presented in this study are contained within the manuscript.
